# Uncertainty-driven mixture convolution and transformer network for remote sensing image super-resolution

**DOI:** 10.1038/s41598-024-59384-x

**Published:** 2024-04-24

**Authors:** Xiaomin Zhang

**Affiliations:** College of Internet of Things and Artificial Intelligence, Fujian Polytechnic of Information Technology, Fuzhou, 350003 Fujian China

**Keywords:** Mathematics and computing, Computer science

## Abstract

Recently, convolutional neural networks (CNNs) and Transformer-based Networks have exhibited remarkable prowess in the realm of remote sensing image super-resolution (RSISR), delivering promising results in the field. Nevertheless, the effective fusion of the inductive bias inherent in CNNs and the long-range modeling capabilities encapsulated within the Transformer architecture remains a relatively uncharted terrain in the context of RSISR endeavors. Accordingly, we propose an uncertainty-driven mixture convolution and transformer network (UMCTN) to earn a performance promotion. Specifically, to acquire multi-scale and hierarchical features, UMCTN adopts a U-shape architecture. Utilizing the dual-view aggregation block (DAB) based residual dual-view aggregation group (RDAG) in both encoder and decoder, we solely introduce a pioneering dense-sparse transformer group (DSTG) into the latent layer. This design effectively eradicates the considerable quadratic complexity inherent in vanilla Transformer structures. Moreover, we introduce a novel uncertainty-driven Loss (UDL) to steer the network’s attention towards pixels exhibiting significant variance. The primary objective is to elevate the reconstruction quality specifically in texture and edge regions. Experimental outcomes on the UCMerced LandUse and AID datasets unequivocally affirm that UMCTN achieves state-of-the-art performance in comparison to presently prevailing methodologies.

## Introduction

The fundamental objective of single image super-resolution (SISR)^1–6^ techniques is the transformation of low-resolution (LR) images, characterized by blurry artifacts, into high-resolution (HR) counterparts replete with intricate textures and crisp edges. SISR techniques have garnered considerable recognition and interest within both academic and industrial, primarily owing to their aptitude in serving as a pre-processing step for a serious of high-level tasks, encompassing domains such as hyper-spectral imaging^7,8^, medical imaging^9,10^, nature imaging^11–13^, and remote sensing imaging^14,15^. Being a classic ill-posed problem, SISR presents a considerable challenge due to the abundance of potential solutions for a given LR input. Besides, owing to the constraints imposed by image transmission conditions (imaging distance, weather conditions, and etc.) and the inherent limitations of imaging equipment (sensor size, aperture size, and etc.), the remote sensing images (RSIs) procured are predominantly Low-Resolution (LR) images marred by undesirable artifacts. These low quality images inevitably degrade the performance of downstream high-level tasks. Hence, the challenge lies in resolving how to effectively enhance the resolution of images, requiring a solution to be addressed. Augmenting the resolution through hardware enhancements proves to be a laborious and economically demanding endeavor. Consequently, the adoption of hardware-agnostic Remote Sensing Image Super Resolution (RSISR) algorithms for enhancing the resolution of RSIs emerges as a viable approach. This approach is progressively assuming a dominant role in the realm of super-resolution reconstruction for RSIs, owing to its formidable reconstruction capabilities and cost-effectiveness.

To improve the resolution of images, researches have proposed a variety of traditional approaches which often leverage various prior knowledge, e.g., sparse prior^16^, low-rank prior^17^ , non-local similarity^18^, and edge prior^19^. Although these methods deliver appreciable performance, their effectiveness hinges largely on the extent of congruence between the manually designed priors and the intricacies of real-world image processing. Furthermore, these approaches frequently entail protracted optimization periods, primarily owing to their constrained synergy with contemporary hardware acceleration resources, such as GPUs. And, these methods are conventionally tailored for addressing non-convex optimization challenges. More importantly, models reliant on manually crafted prior knowledge frequently demonstrate subpar generalization performance, thereby imposing substantial constraints on their practical applicability.

Recent RSISR methods based on CNNs have transitioned away from hand-crafted priors, embracing instead data-driven learning-based priors, which are cultivated through extensive big data sources. Thanks to the impressive feature extraction and fitting capabilities of CNNs, learning-based methods have taken a substantial lead over those of traditional methods. Nonetheless, these methods remain somewhat inadequate in the quest to fully comprehend the intricacies of RSIs, encompassing aspects such as global structures and local finer details. For example, LGCNet^20^ represents the inaugural model built upon CNNs; however, it employs a mere handful of convolution layers for the reconstruction of LR RSIs. This constrained receptive field fails to adequately capture the essential global perspective, particularly significant in the context of remote sensing images that encompass a broad expanse. While HSENET^21^ employs a multi-scale sell-attention mechanism to grasp multi-scale self-similarity and long-range dependency, the self-attention often tends to emphasize low-frequency information, inadvertently neglecting the intricate high-frequency details. This oversight adversely affects the quality of the resultant reconstructed RSIs. Hence, *the challenge of effectively prioritizing both the global structural information and local fine-grained details remains largely unaddressed within the realm of RSISR.*

To deal with the above problems, we propose a pioneering Uncertainty-driven Mixture Convolution and Transformer Network (UMCTN) that adeptly amalgamates the inherent local inductive bias of CNN and the potent non-local dependency modeling capabilities of the Transformer. To intelligently amalgamate the merits of CNNs and ViTs without substantially escalating the computational burden, we employ Residual Dual-view Aggregation Group (RDAG) to efficiently extract local detail information while utilizing Dense-Sparse Transformer Block (DSTB) solely in the latent space to model global structural information and non-local dependencies. In contrast to prior SOTA methods, this design notably diminishes the computational complexity. It’s important to note that with DSTB, self-attention is concurrently computed in both dense and sparse regions. We partition the multi-heads into separate parallel groups and concurrently employ distinct self-attention operations for each group. Through this parallel strategy, each transformer block has an extended scope for self-attention computation, all without incurring additional computational expenses. Besides, grounded in the observation that the texture and edges within an image house significant visual information, in stark contrast to the relatively meager content typically found in smoother regions. Nonetheless, within prevailing RSISR reconstruction algorithms, it is customary to employ $L_{1}$ or Mean Squared Error (MSE) loss, thereby treating each pixel within the image uniformly. Consequently, inspired by^22^, we introduce an uncertainty-driven loss (UDL) for RSISR, facilitating the network’s ability to concentrate on demanding scenarios, such as texture and edge pixels, while incorporating spatial adaptability. To be specific, pixels characterized by heightened certainty will receive preferential treatment during the reconstruction process.

The principal technical contributions of this paper can be summarized as follows:

1. We present a novel Remote Sensing Image Super-Resolution (RSISR) approach, designated as UMCTN. This method incorporates a hybrid architecture that combines Convolutional Neural Networks (CNNs) and Transformer Networks. Additionally, it integrates an adaptive loss mechanism guided by uncertainty.
2. A meticulously designed hybrid feature exploration network is formulated with the dual objectives of adeptly capturing and faithfully restoring high-frequency details in remote sensing images. This design imparts the network with the inductive bias of Convolutional Neural Networks (CNNs) and the capacity to model pairwise long-range dependencies characteristic of Transformer networks.
3. We propose an uncertainty-driven loss, endowing the network with the ability to dynamically focus on intricate, high-frequency regions, thereby conferring spatial adaptability. Moreover, the seamless integration of Uncertainty-Driven Loss (UDL) into any pre-existing Remote Sensing Image Super-Resolution (RSISR) framework enhances reconstruction quality without incurring additional computational costs.
4. UMCTN demonstrates competitive performance across two public datasets, showcasing commendable results in both objective and subjective quality metrics. Comprehensive experiments and ablation studies have substantiated the effectiveness of UMCTN.

## Related works

In this section, we analyze several key approaches relevant to our method, encompassing DL-based Nature Image Super-Resolution, Remote Sensing Image Super-Resolution, and Vision Transformers. Given the exceptional performance demonstrated by DL-based approaches in recent years, our primary focus lies in the introduction of DL-based methods.

### Single nature image super-resolution

DL-based methodologies have dominated the field of Single Image Super-Resolution (SISR) in recent years due to advancements in neural network technologies and the availability of large-scale datasets. Dong et al.^20^ pioneered the initial SISR approach using CNN (SRCNN). Despite having only three convolutional layers, SRCNN surpassed earlier conventional approaches in performance and effectiveness. Dong et al. introduced the seminal SISR approach based on very simple but effective CNNs known as SRCNN^20^. Despite comprising only three convolutional layers, SRCNN outperformed previous conventional approaches in terms of performance and effectiveness. Subsequently, researchers equipped the SISR algorithm with various advanced techniques such as residual connections, dense connections, attention mechanisms, among others, anticipating improved reconstruction performance and yielding promising results. Kim et al. introduced the very deep super-resolution (VDSR) model, leveraging residual connections^23^. With 20 convolutional layers, VDSR significantly outperformed SRCNN, highlighting the positive correlation between increased network depth and enhanced performance in SISR tasks^24^. In the quest for achieving superior performance, investigators persisted in their efforts to develop deeper or more intricate networks. EDSR, proposed by Lim et al.^25^, developed a neural network composed of approximately 50 layers by eliminating redundant subassemblies such as Batch Normalization, aiming to improve the network’s performance in SISR task. However, this approach treats low-resolution (LR) features uniformly, neglecting their long-range correlations, leading to inefficient retrieval of details. Henceforth, a myriad of recent methodologies has surfaced, amalgamating various attention mechanisms into currently modern SR models to recalibrate the significance of diverse elements within the process^26^. Zhang et al. employed a RIR structure in their network (RCAN)^27^ with the aim of enhancing the reconstruction performance. Moreover, RCAN also introduces a channel attention mechanism aimed at augmenting the discriminative representation within the network. Jiang et al.^28^ advocated for a novel hierarchical dense connection network (HDN) designed for image SR, emphasizing a balanced consideration of both reconstruction performance and efficiency. Furthermore, acknowledging the inherent limitations of convolutional networks such as smaller receptive fields, certain researchers have shifted their focus towards the Transformer architecture. This architecture, renowned in NLP, aims to reinvigorate global dependencies within the context of SISR. Lu et al.^29^ employed both the Transformer architecture and CNN structure to construct a lightweight and efficient hybrid network named ESRT. Notably, ESRT introduces an Efficient Multi-Head Attention specifically aimed at minimizing the computational workload involved in capturing long-range pixel interactions. More recently, Cai el al.^30^ developed HIPA leveraging the powerful transformer architecture and achieved better performance.

### Remote sensing image super-resolution

Super-resolution for remote sensing images has become a prominent area of research, showcasing significant advancements and garnering notable attention in recent times. DL-based methods, as highlighted in Salvetti et al.^31^, have notably surpassed the performance of earlier classical methods in this domain. LGCNet, as introduced in Lei et al.^32^, stands as the pioneering DL-based model tailored for RSISR task. Certainly, the incorporation of both local and global representations has proven instrumental in enhancing the learning process for reconstructing high-resolution images. Dong et al. proposed SMSR^33^, a method that aggregates diverse multi-scale and hierarchical representations using first-order and higher-order learning mechanisms. Certainly, over recent years, attention mechanisms have achieved significant advancements in various image analysis tasks, such as remote sensing image classification^34^ and object detection^35^. These mechanisms have contributed notably to enhancing the performance of these tasks. Consequently, attention mechanisms have been developed into the realm of RSISR tasks. HSENet, as outlined in Lei et al.^21^, harnesses single-scale and cross-scale self-similarity information via multi-scale Non-Local attention. Chen et al.^36^ devised a split attention fusion block, enabling the method to accommodate diverse multi-scale land surface reconstructions. Liang et al.^37^ introduced a Multi-scale Hybrid Attention Graph Convolutional Neural Network (MAGSR) tailored for remote sensing image super-resolution (SR). MAGSR aims to extract a broader range of multi-scale deep features and multi-scale high-frequency detail information from the images. Wang et al. introduced a lightweight Feature Enhancement Network (FeNet) aimed at diminishing the memory usage and computational load of the model while enhancing its performance. Moreover, numerous researchers have integrated Generative Adversarial Networks (GANs)^38^ into remote sensing super-resolution tasks to generate visually appealing remote sensing images. Additionally, Li et al.^39^ introduced an attention-based GAN known as SRAGAN, which amalgamates both local and global attention mechanisms. This combined approach aims to discern features of diverse sizes on different objects within the remote sensing images. Xu et al.^40^ devised an improved generative adversarial network dubbed TE-SAGAN. This improved model incorporates self-attention mechanisms and focuses on texture enhancement within the generated images. Jia et al.^41^ designed multi-attention GAN to solve the problem that texture information of various remote sensing images is completely different. More importantly, to address the discrepancy between training data distribution and actual degraded images, Zhao et al.^42^ curated a genuine remote sensing dataset, enabling the training of SR models for authentic scenes. Furthermore, they introduced second-order channel attention to bolster the model’s performance in real-world scenarios.

### Vision transformer

The Transformer architecture^43^ , initially devised for addressing one-dimensional sequence challenges, garnered substantial acclaim upon its introduction and has since established a preeminent foothold within the domain of Natural Language Processing (NLP). The Transformer architecture’s innate capacity for global pixel-by-pixel modeling has prompted numerous researchers to embark on the endeavor of adapting this architecture to computer vision tasks. In a groundbreaking initiative, Vision Transformer (ViT)^44^ undertakes the decomposition of images into one-dimensional tokens, subsequently employing the vanilla self-attention mechanism to attain commendable outcomes in high-level tasks, including image classification^45,46^. The fundamental distinction between vision transformers and CNNs lies in their approach to global context modeling: while CNNs predominantly utilize convolution, vision transformers leverage multi-head self-attention mechanisms. After that, DETR^47^ represents a pivotal milestone in the realm of end-to-end target detection models, leveraging the transformative power of the Transformer architecture for the purposes of object detection. This innovation obviates the need for intricate procedures like anchor frames and Non-Maximum Suppression (NMS) that are customary in traditional target detection methodologies. Consequently, a series of Transformer-based approaches have been introduced, with the overarching goal of enhancing the efficiency and effectiveness of Transformer architectures. Swin Transformer^48^ incorporates a localized windowing mechanism, which serves to confine the scope of attention, thereby effectively mitigating the computational complexity associated with the model. Beyond these high-level tasks, Chen et al.^49^ introduces an innovative pre-training model known as the Image Processing Transformer (IPT). This model exhibits the capability to concurrently address various image restoration tasks, encompassing denoising, de-blurring, detain, super-resolution, and so on. Moreover, Chen et al.^50^ design a dual aggregation transformer (DAT) for image SR, combining the two dimensions in self-attention for a more powerful representation capability. In contrast to the aforementioned models, the primary objective of our proposed model resides in the judicious exploitation of the merits inherent in both CNNs and Transformer networks. This approach is designed to comprehensively apprehend global low-frequency structural information and intricate local high-frequency details, ultimately elevating the network’s prowess in feature representation.

## Methods

This section delineates the overall network architecture of the proposed UMCTN, presenting comprehensive details regarding the Residual Dual-view Aggregation Group, Dense-Sparse Transformer Block, and Uncertainty-driven Loss Function.

**Figure 1:**
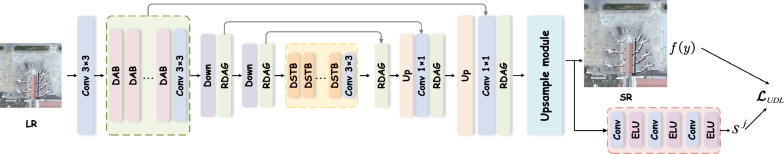
The Illustration of the network architecture of our proposed UMCTN with uncertainty driven loss. UMCTN leverages an encoder-decoder structure. Residual Dual-view Aggregation Group (RDAG) contains *N* Dual-view Aggregation Block (DAB) followed by a Conv layer.

### Overall pipeline

**Figure 2:**
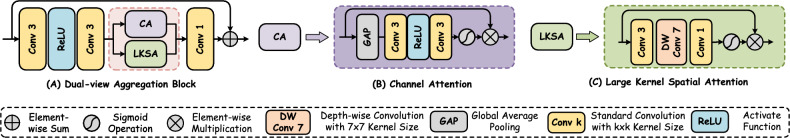
Architecture of the developed Dual-view Aggregation Block (DAB) that consists of four components, i.e., Residual Block, Channel Attention, Large kernel Spatial Attention and one $1 \times 1$convolution layer. Residual Block contains two $3 \times 3$ convolution layers and a ReLU activation function in between.

As illustrated in Fig. 1, the proposed UMCTN adopts the general encoder-decoder structure to learn multi-scale and hierarchical representations efficiently and effectively. Both encoder and decoder block consist of there different spatial resolution scales. More specifically, in both the encoder and decoder sub-networks, there exist three Residual Dual-view Aggregation Groups (RDAG). Within each RDAG submodule contains *N* consecutive cascaded Dual-view Aggregation Blocks (DABs) (as shown in Fig. 2), succeeded by a $3 \times 3$ convolution layer. This arrangement aims to enhance the stability of the network.

Given a degraded low-resolution image $\mathcal {I}_{LR} \in \mathbb {R}^{3 \times H \times W}$, a sole $3 \times 3$ convolution layer is leveraged to explore and investigate shallow and low-frequency features, presenting a size of $C \times H \times W$, where *C* symbolizes the number of channels and $H \times W$ is the spatial locations. Inspired by prior works^27,28^, we believe that a basic 3 x 3 convolution operation is adequate for transitioning features from the image domain to the complex high-dimensional feature domain. Subsequently, the resulting features are directed into three distinct encoder sub-module to acquire multi-scale hierarchical in-depth information. Throughout this process, the channel dimension undergoes doubling, while concurrently, the spatial dimension is reduced by half. Note that, the deepest features with lowest resolution $\frac{H}{4} \times \frac{W}{4}$. Besides, the downsampling operation is executed using a max-pooling operation. Next, the deepest features are channeled through the novel dense-sparse transformer groups to effectively model long-range dependencies and non-local correlations. Performing self-attention computation within the smallest spatial dimension significantly alleviates the computational burden. This approach enables the utilization of self-attention with quadratic complexity on high-resolution images. Then, the consolidated resulting features traverse through the decoder sub-module to recover high-resolution representations. This process employs a transposed convolution with a $4 \times 4$ kernel size to upsample the features. Additionally, the decoder sub-module features are concatenated with encoder sub-module features, following prior practices^51^ to aid in the reconstruction process. Subsequently, a $1 \times 1$ convolutional operation is applied to alter the channels. Finally, a sub-pixel layer is applied to super-resolver to predict the final high-resolution image $\mathcal {I}^{SR} \in \mathbb {R}^{3 \times sH \times sW}$, where *s* symbolizes the upsampling factor.

### Residual dual-view aggregation group (RDAG)

Recently, the visual attention mechanism has garnered significant attention within the computer vision community, particularly concerning low-level image processing tasks. To explore both the channel dimension and the spatial dimension clues, We utilize multiple cascaded Dual-view Aggregation Block (DAB) modules as the fundamental building blocks for both the encoder and decoder, as illustrated in the green box in Fig. 1. DAB utilizes channel attention and large kernel spatial attention, facilitating the extraction of global and local features, leading to precise and efficient restoration of texture details. Specifically, given input feature map $\mathcal {F}_{in}^{RDAG} \in \mathbb {R}^{H \times W \times C}$, we leverage *N* cascaded DAB blocks followed by a $3 \times 3$ Convolution layer to explore and aggregate comprehensive feature representation:

1 $$\begin{aligned} \mathcal {F}_{n} = \mathcal {H}_{3 \times 3}\left( \mathcal {H}_{DAB}^{n}\left( ... \mathcal {H}_{DAB}^{1}\left( \mathcal {F}_{in}^{RDAG}\right) \right) \right) \end{aligned}$$

where $\mathcal {H}_{DAB}^{n}(\cdot )$ and $\mathcal {F}_{n}$ respectively denote the function of *n*-th DAB and its corresponding features, $1 \le n \le N$. $\mathcal {H}_{3 \times 3}$ symbolizes a $3 \ times 3$ convolution operation. Next, we shall elaborate on the precise implementation of the DAB module.

#### Dual-view aggregation block (DAB)

First, we leverage a residual block which contains two $3 \times 3$ convolution layers and a ReLU activation function in between to extract shallow feature representation:

2 $$\begin{aligned} \mathcal {F}^{RB}=\mathcal {H}_{3\times 3}\left( \delta \left( \mathcal {H}_{3\times 3}\left( \mathcal {F}_{in}^{DAB}\right) \right) \right) \end{aligned}$$

Where $\mathcal {F}^{RB}$ denotes the output of the residual block. $\delta (\cdot )$ refers to ReLU activation function. As known, the majority of deep learning-based super-resolution methods often fail to fully leverage the informative features that play a crucial role in the final image recovery process. Hence, we employ a hybrid attention block consists of two parts: (1) channel attention (CA); (2) large kernel attention block. Note that the hybrid attention can focus both on global and local similarity relationships.

**Channel attention** Specifically, we employ a squeeze-and-excitation sub-module^52^, emulating the visual attention mechanism observed in human eyes, to concentrate on the reconstruction process of intricate details. More specifically, we first typically build channel descriptors via a global average pooling. Given input feature $\mathcal {F}^{RB}$, the channel descriptors can be calculated by:

3 $$\begin{aligned} {\mathcal {Z}}_{c}= \frac{1}{H \times W} \sum _{i=1}^{H} \sum _{j=1}^{W}{\mathcal {F}}_{c}^{RB}(i, j) \end{aligned}$$

where $\mathcal {Z}_{c}$ indicates the *c*-ch channel descriptor. Subsequently, we leverage a compact gated sub-block to efficiently redistribute resources via a channel recalibration mechanism.

4 $$\begin{aligned} \mathcal {F}_{out}^{CA} = \mathcal {F}^{RB} \cdot \sigma \left( W_{U}\left( \delta \left( W_{D}({\mathcal {Z}})\right) \right) \right) \end{aligned}$$

where the notation $\mathcal {F}_{out}^{CA}$ represents more comprehensive and information-enriched features following calibration. $W_{U}$ and $W_{D}$ refer to the weights of two $1 \times 1$ convolution layers leveraged to respectively augment and diminish the channel count by a reducing factor. $\sigma (\cdot )$ denote the gate unit (In this paper, we use sigmoid function).

**Large kernel spatial attention (LKSA)** Inspired by the recent advancements in large kernel convolution^53^, we incorporate a $7 \times 7$ deep separable convolution. This choice allows us to effectively gather local detail information, aggregating the hierarchical details inherent in the input images and achieving precise texture detail recovery. Specifically, given an input tensor $\mathcal {F}^{RB} \in \mathbb {R}^{H \times W \times C}$, the LKSA is formulated as:

5 $$\begin{aligned} \mathcal {F}_{out}^{LKSA} = \mathcal {F}^{RB} \cdot \sigma \left( \mathcal {H}_{1 \times 1}\left( \mathcal {H}_{DW 7\times 7}\left( \mathcal {H}_{3 \times 3}\left( \mathcal {F}^{RB}\right) \right) \right) \right) \end{aligned}$$

where $\mathcal {H}_{1 \times 1}(\cdot )$ is the $1 \times 1$ point-wise convolution, $\mathcal {H}_{DW 7\times 7} (\cdot )$ is the $7 \times 7$ depth-wise convolution to explore local details. $\mathcal {H}_{3 \times 3}(\cdot )$ is the $3 \times 3$ convolution. $\sigma (\cdot )$ denotes the gate operation to regulates the flow of information. Note that the LKSA enables each location to capture fine details that complement the channel attention enhanced feature $\mathcal {F}_{out}^{CA}$.

Finally, we leverage a $1 \times 1$ point-wise convolution layer to merge these distinct features ($\mathcal {F}_{out}^{CA}, \mathcal {F}_{out}^{LKSA}$), mitigating any potential feature conflict issues. The fusion process is formulated as:

6 $$\begin{aligned} \mathcal {F}_{out}^{DAB} = \mathcal {H}_{1 \times 1}\left. \left( \mathcal {F}_{out}^{CA} + \mathcal {F}_{out}^{LKSA}\right) \right) + \mathcal {F}_{in}^{DAB} \end{aligned}$$

Here, as to previous SOTA works^26^, we incorporate residual connections, emphasizing learning high-frequency information and enhancing the stability of network training.

### Dense-sparse transformer block (DSTB)

**Figure 3:**
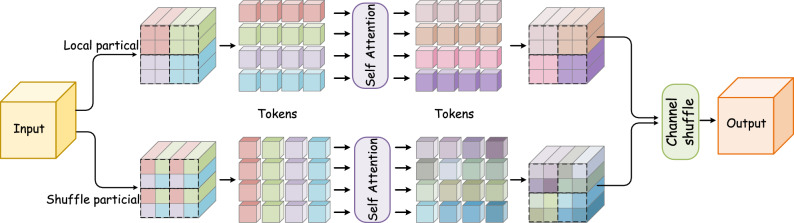
The Illustration of the network architecture of our proposed Dense-Sparse Transformer Block. It consists of a dense self-attention branch and a sparse self-attention branch.

Although possessing a robust capacity for long-range context modeling, the original full self-attention mechanism exhibits quadratic computational complexity concerning the feature map size. Hence, tasks reliant on high-resolution feature maps, like remote sensing image super-resolution, suffer from considerable computational expenses. To mitigate this challenge, prior studies propose conducting self-similarity within a local-region window and implementing a shift operation to expand the receptive field^48^. Nonetheless, employing this operation significantly restricts the global modeling capability inherent in self-attention mechanisms. To broaden the attention span and accomplish global self-attention more efficiently, we develop a novel dense-sparse transformer mechanism, which is realized be perform self-attention in local windows and shuffle windows. As shown in Fig. 1, Transformer sub-module adopts a cascaded structure contained by the basic unit Dense-Sparse Transformer Block (DSTB), as shown in Fig. 3. The input feature map of DSTB are denoted as $\mathcal {F}_{in} \in \mathbb {R}^{C \times H \times W}$. Subsequently, $\mathcal {F}_{in}$ are split into two equal parts along the channel dimension as

7 $$\begin{aligned} \mathcal {X}_{L}, \mathcal {X}_{S} = split(\mathcal {F}_{in}) \end{aligned}$$

**Local partical branch** The local partical branch aggregates features within position-specific local windows. $\mathcal {X}_{L} \in \mathbb {R} ^{\frac{C}{2} \times H \times W}$ is initially partitioned into non-overlapping windows of window size $M \times M$. Then they are reshaped into $\mathbb {R}^{\frac{H \times W}{M \times M} \times M^{2} \times \frac{C}{2}}$. Subsequently, the reshaped feature is linearly projected into $\mathcal {Q}_{L}, \mathcal {K}_{L}, \mathcal {V}_{L}$ as:

8 $$\begin{aligned} \mathcal {Q}_{L}=\mathcal {X}_{L} \cdot \mathcal {W}_{Q}^{L}, \mathcal {K}_{L}=\mathcal {X}_{L} \cdot \mathcal {W}_{K}^{L}, \mathcal {V}_{L}=\mathcal {X}_{L} \cdot \mathcal {W}_{V}^{L} \end{aligned}$$

weher $\mathcal {W}_{Q}^{L}, \mathcal {W}_{K}^{L}, \mathcal {W}_{V}^{L} \in \mathbb {R}^{\frac{C}{2} \times \frac{C}{2}}$ refer to the weight matrix of linear layer. Next, the local window self-attention $\mathcal {A}_{L}$ is calculated inside each window as:

9 $$\begin{aligned} \mathcal {A}_{L} = softmax\left( \frac{\mathcal {Q}_{L} \mathcal {K}_{L}^{T}}{\sqrt{D}} + \mathcal {P}_{L}\right) \end{aligned}$$

where $softmax(\cdot )$ denotes the softmax function, and $\mathcal {P}_{L} \in \mathbb {R}^{M^{2} \times M^{2}}$ denotes the learnable parameters representing the position information. The local enriched feature can be calculated as:

10 $$\begin{aligned} \mathcal {F}_{L} = \mathcal {H}_{3\times 3}(\mathcal {V}_{L} \otimes \mathcal {A}_{L}) \end{aligned}$$

where $\mathcal {F}_{L}$ refers to the enriched local representation. And $\mathcal {H}_{3\times 3}(\cdot )$ denotes the $3 \times$ Convolution layer.

**Shuffle partical branch** The shuffle partical branch explores cross-window collaborations through shuffle operations drawing inspiration from DAUHST^54^. Specifically, $\mathcal {X}_{S} \in \mathbb {R} ^{\frac{C}{2} \times H \times W}$ is also partitioned into non-overlapping windows of window size $M \times M$. Subsequently, their dimensions are reshaped from $\mathbb {R}^{\frac{H \times W}{M \times M} \times M^{2} \times \frac{C}{2}}$ to $\mathbb {R}^{M^{2} \times \frac{H \times W}{M \times M} \times \frac{C}{2}}$ to rearrange the positions of tokens, fostering inter-window relations. Next, the reshaped feature is also linearly projected into $\mathcal {Q}_{S}, \mathcal {K}_{S}, \mathcal {V}_{S}$ as:

11 $$\begin{aligned} \mathcal {Q}_{S}=\mathcal {X}_{S} \cdot \mathcal {W}_{Q}^{S}, \mathcal {K}_{S}=\mathcal {X}_{S} \cdot \mathcal {W}_{K}^{S}, \mathcal {V}_{S}=\mathcal {X}_{S} \cdot \mathcal {W}_{V}^{S} \end{aligned}$$

$\mathcal {W}_{Q}^{S}, \mathcal {W}_{K}^{S}, \mathcal {W}_{V}^{S} \in \mathbb {R}^{\frac{C}{2} \times \frac{C}{2}}$ indicate the weight matrix of linear layer. After that, the global cross window interaction $\mathcal {A}_{S}$ is calculated as:

12 $$\begin{aligned} \mathcal {A}_{S} = softmax\left( \frac{\mathcal {Q}_{S} \mathcal {K}_{S}^{T}}{\sqrt{D}} + \mathcal {P}_{S}\right) \end{aligned}$$

*P*Then, the global enhanced feature can be calculated as follows:

13 $$\begin{aligned} \mathcal {F}_{S} = \mathcal {H}_{3\times 3}\left( \mathcal {V}_{S} \otimes \mathcal {A}_{S}\right) \end{aligned}$$

where $\mathcal {F}_{S}$ refers to the informative global representation. And $\mathcal {H}_{3\times 3}(\cdot )$ denotes the $3 \times$ Convolution layer. Then the outputs of local partical branch and shuffle partical branch are aggregated by a shuffle operation and concat operation as:

14 $$\begin{aligned} \mathcal {F}_{out} = Channel_Shuffle\left( \mathcal {C}\left( \mathcal {F}_{L}, \mathcal {F}_{S}\right) \right) \end{aligned}$$

where $Channel_Shuffle$ and $\mathcal {C}$ indicate channel dimension shuffle and concat operation, respectively.

### Uncertainty-driven loss (UDL)

In our network architecture, the Uncertainty-Driven Loss (UDL) is implemented to augment the efficacy of the network optimization process. We employ $\mathcal {I}_{LR}, \mathcal {I}_{SR}$, and $\mathcal {I}_{HR}$ to represent the low-resolution (LR) image, the recovery high-resolution (HR) image and the corresponding ground-truth (GT) image, respectively. Next, we allow $\mathcal {H}_{SR}(\cdot )$ denotes any super-resolution network. This enables the formulation of the general reconstruction model as follows:

15 $$\begin{aligned} \mathcal {I}_{SR} = \mathcal {H}_{SR}(\mathcal {I}_{LR}) = \mathcal {P}(\mathcal {I}_{HR} | \mathcal {I}_{LR}) \end{aligned}$$

where we expect that the super-resolved $\mathcal {I}_{SR}$ to be as close to the $\mathcal {I}_{HR}$ as possible. The optimization process for super-resolution reconstruction can be defined as maximizing the posterior probability $\mathcal {P}(\mathcal {I}_{HR} | \mathcal {I}_{LR})$. The decomposition of the joint posterior probability into the product of marginals can be achieved by integrating the uncertainty measurement $\Sigma$, expressed as follows:

16 $$\begin{aligned} \mathcal {P}\left( \mathcal {I}_{HR}, \Sigma | \mathcal {I}_{LR}\right) = \mathcal {P}(\Sigma | \mathcal {I}_{LR})\mathcal {P}\left( \mathcal {I}_{HR} | \Sigma , \mathcal {I}_{LR}\right) = \prod p\left( \sigma ^{j} | i_{LR}^{j}\right) p\left( i_{HR}^{j} | \sigma ^{j}, i_{LR}^{j}\right) \end{aligned}$$

where $\sigma ^{j}, i_{LR}^{j},$ and $i_{HR}^{j}$ indicate $j-$th pixel at $\Sigma , \mathcal {I}_{LR}$, and $\mathcal {I}_{HR}$, respectively. Correctly, expressing the aleatoric uncertainty is relatively straightforward, but extracting meaningful conclusions from it remains challenging. Therefore, the marginal probability $\mathcal {P}(\Sigma | \mathcal {I}_{LR})$ cannot be analytically evaluated. To deal with this challenge, we opt to utilize Jeffrey’s prior $p(\sigma ^{j} | i_{LR}^{j}) \approx \frac{1}{\sigma ^{j}}$ based on the assumption that uncertainty tends to exhibit sparsity^22,55^. For the likelihood term $p(i_{HR}^{j} | \sigma ^{j}, i_{LR}^{j})$, our UDL is modeled using Laplace distribution as follows:

17 $$\begin{aligned} p\left( i_{HR}^{j} | \sigma ^{j}, i_{LR}^{j}\right) = \frac{1}{2 \sigma ^{j}} exp\left( - \frac{ |i_{SR}^{j} - i_{HR}^{j}| }{\sigma ^{j}}\right) \end{aligned}$$

where $|\cdot |$ denotes the absolute value operation. Next the maximizing a posteriori estimate problem is what we end up with:

18 $$\begin{aligned} max \sum \left( \ln p\left( \sigma ^{j} | i_{LR}^{j}) + \ln p (i_{HR}^{j} | \sigma ^{j}, i_{LR}^{j}\right) \right) = \underset{i_{SR}^{j},\sigma ^{j}}{\arg \min } \sum \left( e^{-s^{j}} |i_{SR}^{j} - i_{HR}^{j}| + 2s^{j}\right) \end{aligned}$$

where $s^{j}=\ln \sigma ^{j}$, and $\sigma ^{j} = e ^{s^{j}}$. Thus, the ultimate optimization loss for UDL can be defined as follows::

19 $$\begin{aligned} \mathcal {L}_{UDL} = \frac{1}{N} \sum _{n=1}^{N} e ^{- s^{j}}\left( |i_{SR}^{j} - i_{HR}^{j}|\right) + 2 s^{j} \end{aligned}$$

The developed UDL, which bestows upon the network the capability to dynamically concentrate on intricate, high-frequency regions, thereby imparting spatial adaptability to the network. Furthermore, the integration of UDL into any pre-existing Remote Sensing Super-Resolution (RSSISR) framework is seamless, enhancing reconstruction quality without incurring additional computational costs. As shown in Fig. 1, we design an uncertainty block to predict the uncertainty map $s^{j}$.

20 $$\begin{aligned} s^{j}=\delta \left( \mathcal {H}_{Conv}\left( \delta \left( \mathcal {H}_{Conv}\left( \delta \left( \mathcal {H}_{Conv}(\mathcal {I}^{SR}\right) \right) \right) \right) \right) \end{aligned}$$

where $\delta (\cdot )$ denotes the Exponential Linear Unit (ELU) activation function^56^. Besides, $\mathcal {H}_{Conv}(\cdot )$ indicates the convolution layer.

## Experiment

**Figure 4:**
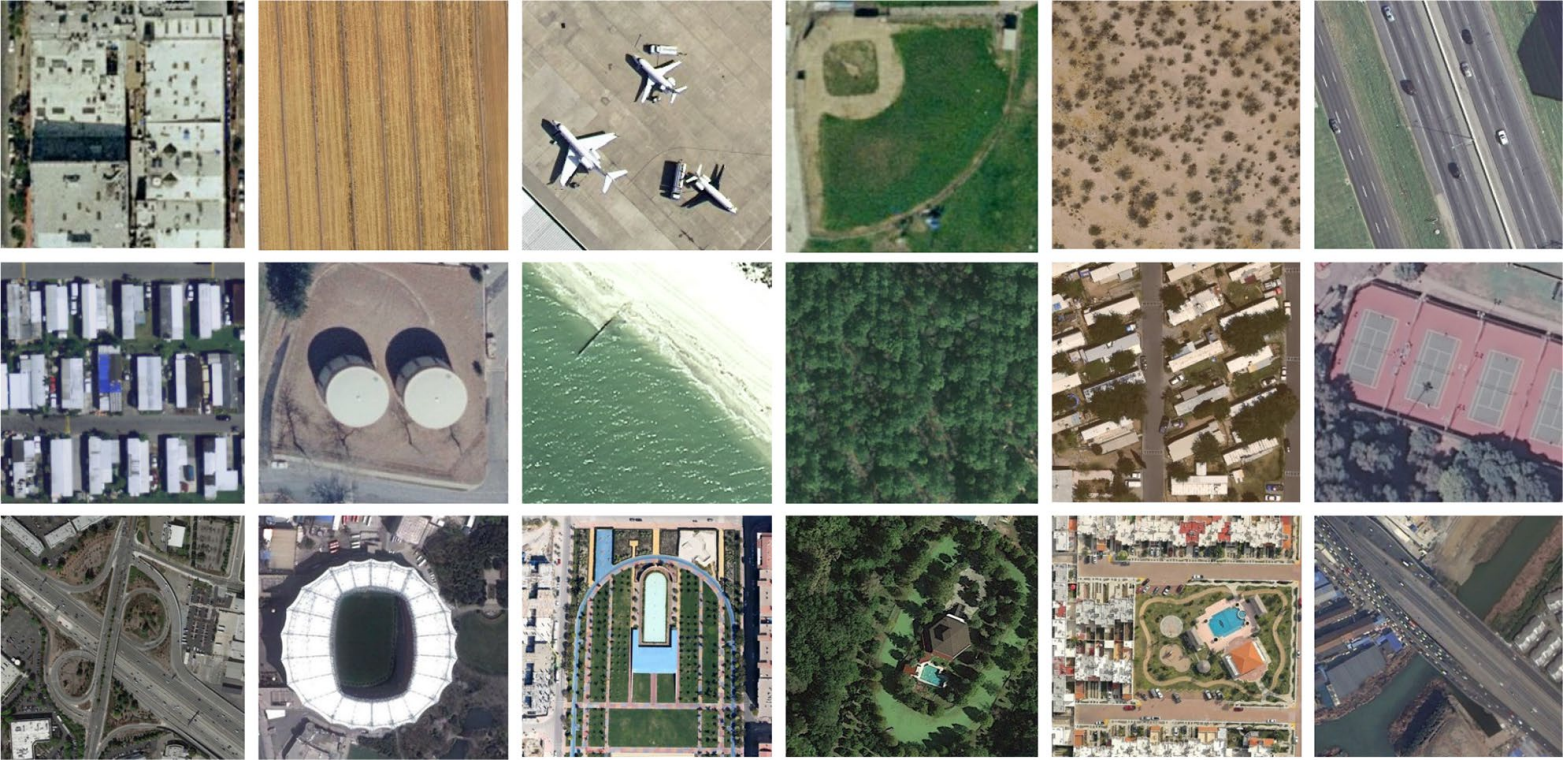
Examples of the different categories of different scenes in the UCMerced LandUse and AID datasets.

### Dataset and metrics

To substantiate the efficacy of our UMCTN method, we employed two widely recognized public remote sensing datasets: UCMerced LandUse^57^ and AID^58^. These datasets hold considerable sway and are frequently employed for appraising RSISR methodologies^21,39,59^. Some examples of these two datasets are shown in Fig. 4. To produce experimentally viable low-resolution images, we conducted downsampling of high-resolution images using scaling factors within the MATLAB environment, utilizing bicubic interpolation.

UCMerced LandUse dataset: This dataset stands as a ubiquitous benchmark in the realm of remote sensing image analysis. It comprises a compendium of 21 distinct categories, housing 100 images within each category, culminating in a total of 2100 images. These categories encapsulate an array of heterogeneous ground image typologies, encompassing urban vistas, agricultural expanses, industrial sectors, and thoroughfares. Each image maintains a pixel resolution of 256x256 and a spatial resolution of 0.3 meters per pixel. In pursuit of constructing a reliable neural network, we partitioned this dataset into training and testing subsets, reserving a subset of 20% from the training set for validation purposes.

AID dataset: This repository stands as a widely embraced resource within the domain of aerial image analysis. It encapsulates a collection of 10,000 images distributed across 30 distinct categories, encompassing various settings such as airports, barren lands, religious edifices, and densely populated urban zones. These images are captured by diverse sensors and from assorted perspectives, averaging around 300-400 images per category. Each image boasts a 600$\times$600 pixel resolution and a spatial resolution of 0.5 meters per pixel. To cultivate a reliable neural network, 80% of the dataset was allocated for training purposes, while the remaining portion was earmarked for testing. Additionally, in a further layer of validation, we systematically selected 5 images at random from each category, amassing a total of 150 images for validation purposes.

Metrics: In assessing the testing dataset against reference images, we perform full-reference evaluations utilizing the Peak Signal-to-Noise Ratio and Structural Similarity Index Measurement (SSIM)^60^ metrics. These metrics gauge the proximity to the reference image, with higher PSNR values indicating closer image content and higher SSIM values reflecting greater similarity in structure and texture. Using the Learned Perceptual Image Patch Similarity (LPIPS)^61^ is a valuable approach to evaluating the reconstruction quality of competing methods. A lower LPIPS value usually indicates higher perceptual quality, as it’s designed to measure perceptual similarity between images. We further conduct an analysis of the FLOPs and parameters of the models to compare the computational complexity among different methods. It’s important to note that the FLOPs are calculated based on a $64\times 64$ image patch size, allowing for a consistent comparison across models.

### Implementation details

To improve generalization performance, we integrate data augmentation techniques that include random rotation , random horizontal flipping, and vertical flipping. These techniques help diversify the training data, enhancing the model’s ability to generalize across various scenarios and inputs. Our implementation and training of the proposed UMCTN are performed using the PyTorch 1.8 framework, leveraging a single NVIDIA RTX A6000 GPU. The Adaptive Moment Estimation Optimizer (ADAM)^62^ with parameters $\beta _{1}=0.9$ and $\beta _{2}=0.999$ is utilized. The learning rate is initially established at $2\times 10^{-4}$, and it undergoes a halving process after every 200 epochs. Throughout the training phase, we extract eight random 64$\times$64 LR patches as a training batch, where the HR image size corresponds to the scaling factor. In addition, 10 DAB modules are included in each encoder or decoder sub-module. There are 5 DSTB modules in the Transformer sub-module.

### Comparisons with the state-of-the-art methods

#### Quantitative results

**Table 1:** The PSNR/SSIM results on UCMerced LandUse Dataset of scale $\times 2$, $\times 3$, and $\times 4$.

Scale	Bicubic	SRCNN	FSRCNN	VDSR	LGCNet	DCM	HSENet	TransENet	Ours
	PSNR/SSIM	PSNR/SSIM	PSNR/SSIM	PSNR/SSIM	PSNR/SSIM	PSNR/SSIM	PSNR/SSIM	PSNR/SSIM	PSNR/SSIM
2	30.76/0.8789	32.84/0.9152	33.18/0.9196	33.38/0.9220	33.48/0.9235	33.65/0.9274	34.22/0.9327	35.43/0.9355	**36.03/0.9821**
3	27.46/0.7631	28.66/0.8038	29.09/0.8167	29.28/0.8232	29.28/0.8238	29.52/0.8349	30.00/0.8420	31.03/0.8526	**31.38/0.8845**
4	25.65/0.6725	26.78/0.7219	26.93/0.7267	26.85/0.7317	27.02/0.7333	27.22/0.7528	27.73/0.7623	28.74/0.7694	**29.22/0.8048**

The best and second results are bold and underlined.

**Table 2:** The PSNR/SSIM results on AID Dataset of scale x2, x3, and x4.

Scale	Bicubic	SRCNN	FSRCNN	VDSR	LGCNet	DCM	HSENet	TransENet	Ours
	PSNR/SSIM	PSNR/SSIM	PSNR/SSIM	PSNR/SSIM	PSNR/SSIM	PSNR/SSIM	PSNR/SSIM	PSNR/SSIM	PSNR/SSIM
2	32.39/0.8906	34.49/0.9286	34.73/0.933	35.05/0.9346	34.80/0.9320	35.21/0.9366	35.24/0.9368	35.28/0.9374	**37.29/0.9688**
3	29.08/0.7863	30.55/0.8372	30.98/0.840	31.15/0.8522	30.73/0.8417	31.31/0.8561	31.39/0.8572	31.45/0.8595	**33.23/0.8899**
4	27.30/0.7036	28.40/0.7561	28.77/0.772	28.99/0.7753	28.61/0.7626	29.17/0.7824	29.21/0.7850	29.38/0.7909	**30.85/0.8193**

The best and second results are bold and underlined.

**Table 3:** Mean PSNR (dB) of each class for upscaling factor 4 on aid test dataset.

Class	Bicubic	SRCNN	LGCNet	VDSR	DCM	HSENet	TransENet	Ours
	PSNR	PSNR	PSNR	PSNR	PSNR	PSNR	PSNR	PSNR
airport	27.03	28.17	28.39	28.82	28.99	29.03	29.23	**29.56**
bareland	34.88	35.63	35.78	35.98	36.17	36.21	36.20	**36.34**
baseballfield	29.06	30.51	30.75	31.18	31.36	31.23	31.59	**31.58**
beach	31.07	31.92	32.08	32.29	32.45	32.76	32.55	**33.49**
bridge	28.98	30.41	30.67	31.19	31.39	31.30	**31.63**	31.52
center	25.26	26.59	26.92	27.48	27.72	27.84	**28.03**	27.90
church	22.15	23.41	23.68	24.12	24.29	24.39	24.51	**24.72**
commercial	25.83	27.05	27.24	27.62	27.78	27.99	27.97	**28.52**
denseresidential	23.05	24.13	24.33	24.70	24.87	24.44	**25.13**	24.94
desert	38.49	38.84	39.06	39.13	39.27	39.37	**39.31**	39.28
farmland	32.30	33.48	33.77	34.20	34.42	33.90	**34.58**	34.43
forest	27.39	28.15	28.20	28.36	28.47	38.31	**28.56**	**28.75**
industrial	24.75	26.00	26.24	26.72	26.92	26.99	27.21	**27.41**
meadow	32.06	32.57	32.65	32.77	32.88	32.74	32.94	**33.42**
mediumresidential	26.09	27.37	27.63	28.06	28.25	28.11	**28.45**	27.21
mountain	28.04	28.90	28.97	29.11	29.18	29.26	**29.28**	29.15
park	26.23	27.25	27.37	27.69	27.82	28.23	28.01	**28.68**
parking	22.33	24.01	24.40	25.21	25.74	26.17	**26.40**	**26.46**
playground	27.27	28.72	29.04	29.62	29.92	31.18	30.30	**32.31**
pond	28.94	29.85	30.00	30.26	30.39	30.40	30.53	**30.67**
port	24.69	25.82	26.02	26.43	26.62	26.92	26.91	**27.25**
railwaystation	26.31	27.55	27.76	28.19	28.38	28.47	**28.61**	28.33
resort	25.98	27.12	27.32	27.71	27.88	27.99	**28.08**	27.72
river	29.61	30.48	30.60	30.82	30.91	30.88	**31.00**	30.83
school	24.91	26.13	26.34	26.78	26.94	27.51	27.22	**27.52**
sparseresidential	25.41	26.16	26.27	26.46	26.53	26.44	26.43	**26.64**
square	26.75	28.13	28.39	28.91	29.13	29.05	**29.39**	28.92
stadium	24.81	26.10	26.37	26.88	27.10	27.28	27.41	**27.73**
storagetanks	24.18	25.27	25.48	25.86	26.00	26.07	26.20	**26.59**
viaduct	25.86	27.03	27.26	27.74	27.93	28.12	**28.21**	28.15
AVG	27.30	28.40	28.61	28.99	29.17	29.21	29.38	**30.95**

Significant values are bold.

The prevailing cutting-edge methodologies, including Bicubic, SRCNN^20^, FSRCNN^63^, VDSR^24^, LGCNet^32^, DCM^64^, HSENet^21^, and TransENet^59^,), have showcased formidable prowess within the realm of image super-resolution. To ascertain the effectiveness of UMCTN, we engaged in an intense comparative evaluation against these eight methodologies. These methods are evaluated quantitatively and visually on the UCMerced LandUse and AID datasets. It is essential to note that all the comparison methods are analyzed using open-source code and trained and evaluated under the same experimental environment. The findings presented in Table 1, showcasing the average results of various methods on the UCMerced LandUse test dataset, distinctly demonstrate that UMCTN surpasses other advanced methods by a considerable margin. UMCTN exhibits superior restoration outcomes across all three upscale factors, presenting the best performance among the evaluated approaches. In specific terms, our model showcases a noteworthy improvement over the second-best method (TransENet) with enhancements of 0.6 dB, 0.35 dB, and 0.48 dB across all three upscale factors in terms of PSNR, respectively. Additionally, concerning the SSIM metric, our model surpasses TransENet by margins of 0.0466, 0.0319, and 0.0354, respectively. Notably, the complexity of UMCTN is only 20% compared to TransNet, primarily attributed to our network’s adeptness in fully harnessing and exploring local detail information and global structure. The AID dataset serves as an additional evaluation benchmark to further assess the generality and generalization performance. This dataset is chosen because the images it contains encompass a wider range of categories and exhibit higher diversity compared to those found in the UCMerced Landuse dataset. The findings in Table 2 clearly demonstrate that UMCTN attains the highest average PSNR and SSIM scores across all three upscale factors. Specifically, in comparison to the current leading method, TransENet, we achieve notable improvements in PSNR and SSIM scores. For upscale factor 2, we enhance the PSNR from 35.28 to 37.29 and the SSIM from 0.9374 to 0.9688. Similarly, for upscale factor 4, we improve the PSNR from 29.38 to 30.85 and the SSIM from 0.7909 to 0.8193. The results demonstrate that, across various scenarios, the devised UMCTN consistently surpasses the performance of existing leading approaches. This reaffirms the superior generalization ability inherent in UMCTN. More importantly, Table 3 presents a comprehensive analysis of various approaches across all 30 scene classes in the AID dataset at a scale factor of 4. UMCTN demonstrates superior PSNR scores in 19 scene classes, outperforming TransENet. Notably, UMCTN achieves an average improvement of 1.57 dB over TransENet, further affirming the effectiveness of our proposed approach.

**Table 4:** The LPIPS results on UCMerced LandUse Dataset of scale x2, x3, and x4.

Scale	Bicubic	SRCNN	FSRCNN	VDSR	LGCNet	DCM	HSENet	TransENet	Ours
	LPIPS	LPIPS	LPIPS	LPIPS	LPIPS	LPIPS	LPIPS	LPIPS	LPIPS
2	0.0721	0.0444	0.0471	0.0287	0.0293	0.0284	0.0266	0.0279	**0.0254**
3	0.1281	0.0945	0.1062	0.0801	0.0752	0.0698	0.0654	0.0649	**0.0644**
4	0.1650	0.1260	0.1395	0.1102	0.1093	0.1046	0.1081	0.1030	**0.1013**

The best and second results are bold and underlined.

#### Perceptual metric

LPIPS, being more aligned with human judgments of image quality compared to other metrics like PSNR or SSIM, is employed to evaluate the quality of super-resolution remote sensing images. We present the LPIPS measure between our UMCTN and state-of-the-art techniques in Table 4. It is evident that when compared to alternative methods, the suggested model produces inferior results-lower is preferable. This illustrates how the proposed UMCTN can produce more realistic and visually satisfying outcomes.

#### Visual comparison

**Figure 5:**
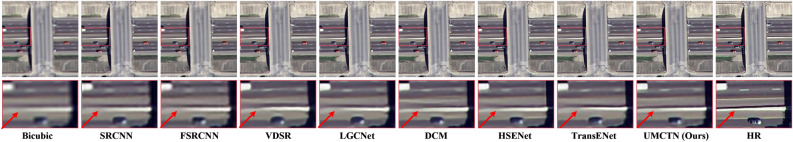
Visual comparison on UCMerced LandUse dataset with scale factor 2.

**Figure 6:**
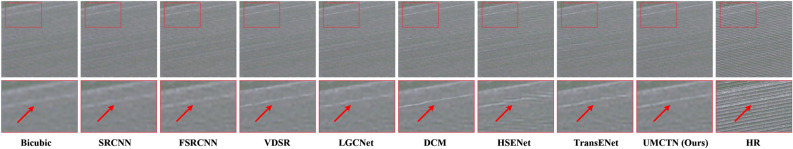
Visual comparison on UCMerced LandUse dataset with scale factor 3.

**Figure 7:**
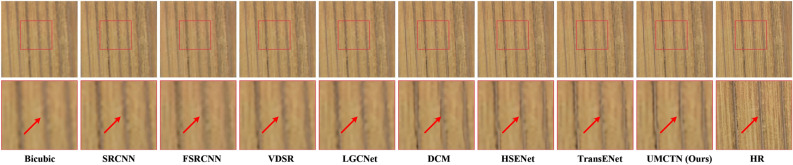
Visual comparison on UCMerced LandUse dataset with scale factor 3.

**Figure 8:**
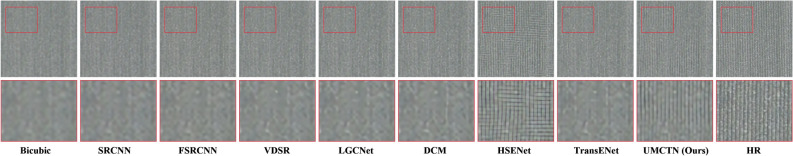
Visual comparison on UCMerced LandUse dataset with scale factor 4.

**Figure 9:**
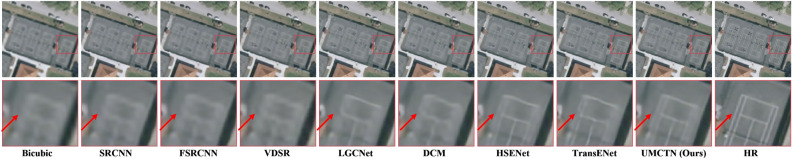
Visual comparison on UCMerced LandUse dataset with scale factor 4.

To further validate UMCTN’s efficacy, we compare it with current emerging approaches. Figures 5, 6, 7, 8 and 9 present multiple example super-resolution results derived from the test set using various approaches, showcasing high-resolution (HR) images. Additionally, a red rectangle denotes a close-up area beneath each image for easy comparison. As depicted in Figure 5 , the Traffic Index Line reconstructed by UMCTN appears clearer and better aligned with the HR requirements. Moreover, UMCTN exhibits more pronounced recovery of details and structural elements, highlighting its improved performance in restoring fine details and structures. As illustrated in Figs. 6, 7 and 8, UMCTN generates the clearest depiction of farmland at higher magnification, surpassing other methods that exhibit varying degrees of blurring, distortion, and warping. This observation also substantiates the advantage of our UMCTN in generating high-quality results compared to others. As depicted in Fig. 9 , the tennis court reconstructed by the suboptimal network suffers from significant loss of lines. In contrast, UMCTN produces an image closest to the HR image, preserving more details and lines, showcasing its superior performance in retaining fine details and structure compared to the suboptimal network. Based on the analysis presented above, it can be concluded that UMCTN demonstrates the capability to generate visually appealing high-resolution images. These images exhibit rich, realistic textures, sharp edges, and distinct boundaries, contributing to their overall visual satisfaction.

### Ablation study

To comprehensively comprehend the performance of the developed UCMTN, an extensive ablation study is conducted, involving in-depth evaluations of each individual module. The ablation investigation is intended to offer additional understanding about the performance of the designed model.

#### Efficacy of our devised DSTB

**Table 5:** Quantitative comparison of different transformer structure on the UCMerced LandUse dataset.

Method	Params	PSNR (dB)	SSIM
Convolution	2.12M	38.23	0.9814
Dense Self-Attention^65^	2.08M	38.71	0.9822
Sparse Self-Attention^65^	2.07M	38.69	0.9821
Dense-Sparse Self-Attention (sequence)^65^	2.24M	38.69	0.9822
DSTB (Ours)	2.12M	**38.76**	**0.9826**

The best results are in bold.

Table 5 displays the outcomes of several ablation experiments conducted to validate the efficacy of the proposed DSTB. It is worth noting that we use pure convolution-based network as the baseline model. To ensure a fair comparison, model parameters using different architectures were constrained to the same level. Should we refrain from utilizing any self-attention mechanism, the reconstruction performance will experience a substantial decline. The inclusion of dense self-attention notably enhances the PSNR and SSIM scores by 0.48 dB and 0.0008, respectively. This enhancement can be attributed to the self-attention’s inherent global modeling capability and its capacity to capture non-local features. These attributes contribute to bolstering the global structure of the model and augmenting the extraction of low-frequency information. In addition, Sparse Self Attention achieves similar performance, but the reconstruction performance is hampered by the lack of focusing on features within the window. Thus, we employ both dense self-attention and sparse self-attention mechanisms to absorb both intra-window and inter-window information effectively. An intuitive approach might involve executing dense self-attention and sparse self-attention consecutively. However, indiscriminate utilization of these two distinct attention mechanisms leads to significant differences in the produced features. Consequently, such an approach results in a collapse in the model’s performance, manifesting as a 0.02 dB performance degradation, as indicated in the Table 5 . Consequently, we developed an adaptive parallel module named DSTB . This module is designed to autonomously learn and integrate the distinct features from both dense and sparse attention mechanism.

#### Effectiveness of hybrid architecture

**Table 6:** Quantitative comparison of different network structure on the UCMerced LandUse dataset.

Method	Params	FLOPs	Memory	PSNR (dB)	SSIM
Pure convolution	2.12M	2.39G	4.2 G	38.23	0.9814
Pure self-attention	2.08M	17.8G	32 G	38.73	0.9823
DSTB (Ours)	2.12M	3.15G	9.7 G	**38.76**	**0.9826**

The best results are in bold.

To affirm the efficacy and advantage of the hybrid architecture developed in this paper, we compare it with both the pure CNN-based model and the pure self-attention-based model. The results of this comparison are presented in Table 6. It’s important to note that, to ensure fairness in the comparison experiments, an encoder-decoder mechanism is employed for all three models, and the model parameters are maintained consistently across the board. As observed, the convolutional neural networks showcase the poorest performance due to the inductive bias features they possess (e.g., translation invariance and parameter sharing, etc.). Conversely, models built on self-attention architectures can readily access global dependencies, thereby enhancing the model’s performance. However, this performance enhancement comes at a considerable cost to model efficiency. In particular, the model based on the Transformer architecture demands 17.8 G of FLOPs and occupies 32 G of GPU memory footprint, yet it achieves a mere 0.5 dB performance gain. We consider this inefficiency to be highly impractical and unsustainable. To tackle this issue, we devise a hybrid model strategy. This approach incorporates the transformer architecture exclusively in the lowest-resolution feature space while employing more efficient convolutional operations in other feature spaces. As depicted in the Table 6, our proposed hybrid architecture model effectively harnesses both convolutional networks and self-attention capabilities without imposing a significant additional computational burden. This amalgamation leads to improved reconstruction performance in the model.

#### Effectiveness of our proposed UDL

**Table 7:** Quantitative comparison of different loss functions on the UCMerced LandUse dataset.

Method	Training Time (s)	PSNR (dB)	SSIM
*L*1 Loss	0.7	38.71	0.9822
*L*2 Loss	0.7	38.69	0.9821
Two-stage UDL^22^	1.5	**38.77**	**0.9826**
One-stage UDL (Ours)	0.8	38.76	0.9826

The best results are in bold.

The purpose of this section is to present findings on the influence of different loss functions on reconstruction performance. Initially, we select two commonly used loss functions, namely *L*1 and *L*2 loss, for comparison purposes. This is intended to showcase the superiority and effectiveness of our proposed UDL function in RSSISR tasks. The quantitative comparisons are shown in Table 7. The quantitative comparisons are shown in Table 7. It is found that our method obtains a better reconstruction performance when leveraging the proposed UDL loss function. In contrast to the model utilizing L1 loss, the model employing UDL showcases enhancements of 0.05 dB and 0.004 in PSNR and SSIM metrics, respectively. Similarly, when compared to the model using L2 loss, the UDL-based model demonstrates enhancements of 0.07 and 0.005 in PSNR and SSIM metrics, respectively. We attribute this phenomenon to the fact that the *L*1 loss function does not penalize large errors adequately, while the *L*2 loss function tends to converge slowly. Therefore, we advocate prioritizing pixels that display high variance in low-level and ill-posed RSISR tasks. This prioritization is crucial as it significantly enhances the quality of the reconstruction process. Moreover, the principal distinction between the Uncertainty-Driven Loss (UDL) proposed in this paper and the approach in Ref.^22^ lies in the adoption of a one-stage training method for the UDL loss function in our proposal. This allows seamless integration with existing state-of-the-art (SOTA) models and significantly reduces the time required for model training. Specifically, the time required using a two-stage training strategy is almost double that of a one-stage strategy. Additionally, we observe that the use of a single-stage training strategy has minimal impact on performance. Therefore, this paper ultimately adopts a single-stage training strategy.

#### Effectiveness of DAB

**Table 8:** Ablation studies of different components in DAB on the UCMerced LandUse dataset.

Method	Params	PSNR (dB)	SSIM
Residul Block	2.02M	38.23	0.9814
W/ CA	2.05M	38.28	0.9817
W/ LKSA	2.08M	38.31	0.9818
W/ CA + LKSA	2.12M	38.34	0.9820

The best results are in bold.

To further validate the efficacy of our proposed Dual-view Aggregation Block (DAB), we conducted a series of ablation experiments, and the outcomes are delineated in Table 8. Initially, we utilized a pure Residual Block (RB) based network as the baseline. Subsequently, we made continuous modifications to the corresponding module to verify the efficacy of the proposed sub-modules. As we can see, the inclusion of the CA mechanism results in a noticeable enhancement of 0.05 dB in PSNR performance and a 0.0003 improvement in SSIM performance. This improvement can be primarily attributed to the CA mechanism’s ability to recalibrate features, suppress irrelevant information, and prioritize information-rich features, optimizing the utilization of computational resources effectively. Merely delving into the channel-wise cues of the network does not fully exploit the hierarchical relationship within the input representation. Consequently, we introduce the LKSA sub-module, empowering the network to concentrate on information-rich regions. As shown in the TABLE, LKSA led to an improvement of 0.08 dB in PSNR and 0.004 in SSIM. This enhancement is attributed to its larger receptive field, allowing the network to explore finer details. Subsequently, merging the features from these distinct perspectives enhances the network’s ability to aggregate richer hierarchical information, thereby boosting the reconstruction performance. These comparisons undeniably highlight the effectiveness of our proposed sub-modules.

### Model complexity analysis

**Figure 10:**
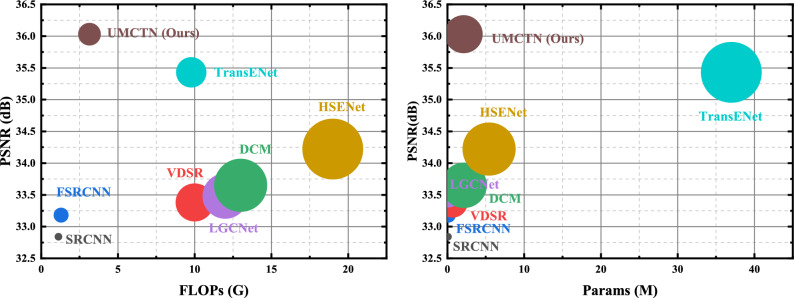
Performance and complexity. Results are evaluated on UCMerced LandUse dataset with scale factor 2. The size of the circle is proportional to the model complexity (e.g., FLOPs and number of parameters).

Figure 10 compares the developed UMCTN model to presently emerging competitors on the UCMerced LandUse dataset in terms of network FLOPs and network parameters. The model size is determined by its parameters, while FLOPs refer to the number of floating point operations needed for computation All methods are measured with the same parameters on a single NVIDIA A6000 GPU to provide a fair comparison. On one hand, our proposed method, UMCTN, achieves superior results with fewer parameters and FLOPs for each upscale factor when compared to other SOTA models such as HSENet and TransENet, suggesting a reasonable balance between complexity and performance. Specifically, our model requires only 20% of the Parameters and 30% FLOPs of the second-best model, while producing a remarkable improvement of 0.604 dB. These findings indicate that UMCTN effectively enables small models to obtain a global receptive field, leading to more efficient recovery of degraded RS images.

## Conclusion

In this article, a brand-new Uncertainty-driven Mixture Convolution and Transformer Network, referred to as UMCTN, is presented for the task of accurate remote sensing image super-resolution (RSISR), which is effective and computationally efficient. The core idea of our work is to simultaneously focus on the local detail information and global structure dependencies. To this end, we propose two modules: Residual Dual-view Aggregation Group (RDAG) and Dense-Sparse Transformer Group (DSTG). RDAG is built on convolution attention layer to detect local detail information for subsequent high-frequency enhancement. Furthermore, DSTG adeptly aggregate global correlation and augments the network’s capacity to discern low-frequency component, thereby complementing RDAG. To reduce the computational complexity of the network, we use a U-shape architecture with RDAG modules in the shallow blocks and DSTG in the deep blocks. More importantly, we introduce a pioneering uncertainty-driven adaptive loss mechanism, designed to train the network to prioritize challenging scenarios, including textures and edges. This innovation serves to elevate the quality of reconstruction in intricate regions. Benefiting from these subassemblies, UMCTN adeptly captures global, long-range, and local relationships in an efficient and effective fashion.Comprehensive experimentation conducted on these public datasets demonstrates that UMCTN surpasses other currently preeminent approaches in both quantitative and qualitative assessments. In the future, we seek to focus our efforts on developing a more general and effective remote-sensing image reconstruction model. Notably, the proposed model is primarily intended for use in processing remote-sensing images; applying it to other settings, such as medical imaging, hyper-spectral images, and so on, is an issue that requires further investigation.

## Data Availability

The datasets used during the study are available from the corresponding author upon reasonable request.
